# NIN-like proteins (NLPs) as crucial nitrate sensors: an overview of their roles in nitrogen signaling, symbiosis, abiotic stress, and beyond

**DOI:** 10.1007/s12298-024-01485-y

**Published:** 2024-07-18

**Authors:** Mariana López Sámano, Kalpana Nanjareddy, Manoj-Kumar Arthikala

**Affiliations:** Ciencias Agrogenómicas, Escuela Nacional de Estudios Superiores Unidad León, Universidad Nacional Autónoma de México (UNAM), 37689 León, Mexico

**Keywords:** Nitrogen, RWP-RK proteins, NIN-like proteins, NLP genes, Symbiotic nitrogen-fixation, Nitrate sensor

## Abstract

Nitrogen is an essential macronutrient critical for plant growth and productivity. Plants have the capacity to uptake inorganic nitrate and ammonium, with nitrate playing a crucial role as a signaling molecule in various cellular processes. The availability of nitrate and the signaling pathways involved finely tune the processes of nitrate uptake and assimilation. NIN-like proteins (NLPs), a group of transcription factors belonging to the RWP-RK gene family, act as major nitrate sensors and are implicated in the primary nitrate response (PNR) within the nucleus of both non-leguminous and leguminous plants through their RWP-RK domains. In leguminous plants, NLPs are indispensable for the initiation and development of nitrogen-fixing nodules in symbiosis with rhizobia. Moreover, NLPs play pivotal roles in plant responses to abiotic stresses, including drought and cold. Recent studies have identified NLP homologs in oomycete pathogens, suggesting their potential involvement in pathogenesis and virulence. This review article delves into the conservation of RWP-RK genes, examining their significance and implications across different plant species. The focus lies on the role of NLPs as nitrate sensors, investigating their involvement in various processes, including rhizobial symbiosis in both leguminous and non-leguminous plants. Additionally, the multifaceted functions of NLPs in abiotic stress responses, developmental processes, and interactions with plant pathogens are explored. By comprehensively analyzing the role of NLPs in nitrate signaling and their broader implications for plant growth and development, this review sheds light on the intricate mechanisms underlying nitrogen sensing and signaling in various plant lineages.

## Introduction

Nitrogen (N) is a crucial macronutrient essential for plant growth, yet it remains limited in availability (Crawford and Glass 1998). As a major component of proteins, chlorophyll, nucleotides, and hormones, nitrogen profoundly influences plant growth and productivity. Plants have the ability to absorb inorganic nitrate in two forms: nitrate (NO_3_^−^) and ammonium (NH_4_^+^). Insufficient nitrogen levels can lead to restricted plant growth and a shift from the vegetative to the reproductive phase. Interestingly, nitrogen deficiency encourages extensive root ramification, while sufficient nitrogen promotes resource allocation for enhanced vegetative growth. The process of nitrogen allocation is heavily influenced by nitrate transporters, which play a vital role depending on nitrogen availability. This highlights the crucial role of nitrate signaling in coordinating nitrogen utilization and plant growth. Additionally, nitrate signaling triggers the activation of genes associated with various metabolic pathways and developmental processes (Zhao et al. 2018).

Unlike most other plants, legumes acquire nitrogen through two distinct pathways. Firstly, they uptake mineral nitrogen from the soil. Secondly, legumes establish a symbiotic relationship with nitrogen-fixing bacteria called rhizobia. This symbiotic interaction leads to the formation of nodules on the roots of leguminous plants, providing a specialized environment for rhizobia. Inside these nodules, rhizobia perform the essential biological process of nitrogen-fixation, converting atmospheric nitrogen (N_2_) into a biologically useful form for plants. As a result, leguminous plants benefit significantly from this nitrogen-fixing capability, which ultimately enhances their growth and overall nitrogen nutrition (Murray et al. 2017).

The initiation of the legume-rhizobia symbiosis occurs in response to nitrogen deficiency. When legume roots experience this deficiency, they release flavonoid molecules into the rhizosphere, prompting compatible rhizobia to synthesize lipochitin oligosaccharide signaling molecules known as Nod factors (NFs) (Sanjuan et al. 1992). These NFs are then perceived by LysM receptors located in the plasma membrane of root epidermal cells. This recognition event triggers the activation of downstream transcription factors, which include *NODULE INCEPTION* (*NIN*), *ETHYLENE-RESPONSIVE FACTOR REQUIRED FOR NODULATION 1* (*ERN1*), and two GRAS proteins, *NODULATION SIGNALING PATHWAY* 1 and 2 (*NSP1* and *NSP2*) (Kalo et al. 2005; Smit et al. 2005; Kawaharada et al. 2017). As a result of this signaling cascade, nodulation genes are activated, initiating the infection process and the formation of nodule primordia. However, the formation of nodules and nitrogen fixation are energy-demanding processes, prompting legumes to evolve an intricate system to regulate both nodule numbers and symbiotic nitrogen-fixation levels. Studies involving split-root experiments have revealed the existence of a root-shoot–root long-distance feedback mechanism known as ´autoregulation of nodulation´, which serves as a control mechanism for regulating nodule number (Magori et al. 2009; Kouchi et al. 2010). This feedback system helps legumes balance the investment in nodules and nitrogen fixation with the overall energy availability and metabolic demands of the plant (Streeter and Wong 1988).

Although the presence of elemental nitrate is known to be a factor in inhibiting nodulation, the precise mechanism responsible for this inhibition has not been fully elucidated yet. In this direction, several recent works enlist several genes associated with nitrate sensing and signaling through molecular and genetic analyses. The proteins encoded by these include *NITRATE TRANSPORTERS* (NRT1.1), *CALCINEURIN-B-LIKE INTERACTING PROTEIN KINASES* (CIPKs), nitrate regulatory proteins, and several transcription factors (TFs) such as ANR1, LBD37/38/39, TCP20, SPL9, NLPs and so on. In this direction, *NIN-LIKE PROTEINS* (NLPs) have emerged as key players in the nitrate signaling pathway not only in nodulating legumes but also in non-legumes (Guan et al. 2014; Vidal et al. 2015; O’Brien et al. 2016; Xu et al. 2016; Roy et al. 2020).

NIN protein, known as the founding member of NIN-like proteins (NLPs), is characterized by the presence of the RWP-RK domain in addition to the PB1 domain (Yokota and Hayashi 2011). Due to the existence of the PB1 domain, NIN proteins in legumes are also categorized as NLPs (Chardin et al. 2014). Interestingly, while NIN is specific to legumes, NLPs have been identified in non-legumes such as rice, *Arabidopsis*, wheat, and maize (Schauser et al. 1999; Konishi and Yanagisawa 2013a, b; Chardin et al. 2014; Kumar et al. 2018).

**Fig. 1:**
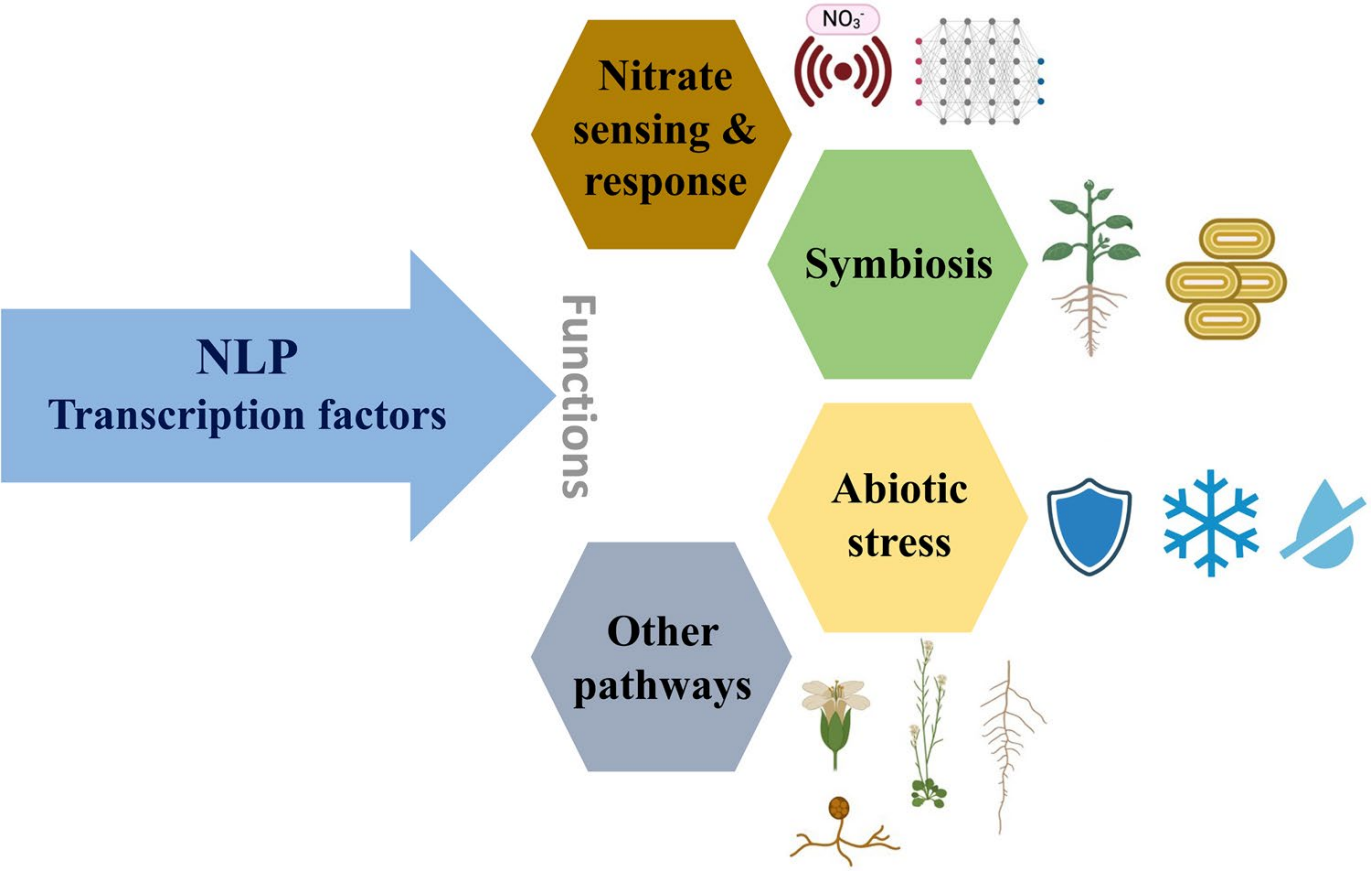
An overview illustrating the functions of NLP transcription factors across multiple processes in nitrate signaling, symbiosis, responses to abiotic stress, and other associated functions

This review article will delve into several key aspects (Fig. 1). Firstly, we will explore the conservation of RWP-RK genes, examining their significance and implications across different plant species. Secondly, we will investigate the role of NLPs as essential nitrate sensing and response, focusing on their involvement in various processes, including rhizobial symbiosis, in both leguminous and non-leguminous plants. By thoroughly examining these topics, we aim to shed light on the intricate mechanisms underlying nitrogen signaling and its broader implications for plant growth and development.

## RWP-RK genes are conserved across plants

In recent years, RWP-RK proteins have emerged as crucial components in the field of nitrate sensing and signaling. Extensive genome-wide investigations have led to the identification of RWP-RK proteins, also known as NIN-like proteins, in numerous plant species (Schauser et al. 2005; Koi et al. 2016; Ge et al. 2018; Kumar et al. 2018; Liu et al. 2018; Wang Z. et al. 2018; Mu and Luo 2019). RWP-RK proteins, characterized by the presence of RWP (Arg-Trp-Pro) and RK (Arg-Lys) motifs, represent a family of transcription factors found in all vascular plants, green algae, and slime molds. They are recognized as a plant-specific transcription factor family (Mu and Luo 2019). The RWP-RK domain is responsible for DNA binding and is defined as a novel class of transcription factors (Schauser et al. 1999; Chardin et al. 2014; Mu and Luo 2019; Yin et al. 2020). This family of RWP-RK proteins consists of two major classes: RWP-RK domain proteins (RKDs) and NIN-like proteins (NLPs).

The RWP-RK domain, spanning 60 amino acids, contains two α-helices and an amphipathic leucine zipper, with the conserved sequence Arg–Trp–Pro–X–Arg–Lys contributing to its DNA binding function (Mu and Luo 2019). TFs containing the RWP-RK domain were initially grouped as one family. However, RKDs have been more recently subdivided into four sub-groups, including RWP-A, RWP-B, RKD, and Minuce Dominance (MID). The latter group, MID, shares a high degree of sequence similarity with the NIN gene and features the PB1 domain (Phox and Bem 1) at their C-termini, along with the RWP-RK domain and GAF-like domain at their N-termini (Wu et al. 2020).

While RKDs function in non-legume plants like *Arabidopsis thaliana*, where they are mainly expressed in reproductive organs and play a role in female gametophyte development (Tedeschi et al. 2017), NIN and NLPs are pivotal in nitrogen-fixation, nitrate signaling pathways, and symbiotic signaling pathways (Suzuki et al. 2013; Hsin et al. 2021). These proteins play essential roles in coordinating nitrogen utilization and plant growth, making them crucial players in understanding plant responses to nitrogen availability and other related physiological processes. The RKD sub-group contains an unnamed conserved region upstream from the RWP-RK domain in all N-termini. The species in this sub-group belong to bryophytes and vascular plants without algal members. The RKD sub-group proteins are functionally associated with egg cell differentiation, specification, and gametophyte development (Rövekamp et al. 2016; Tedeschi et al. 2017). Finally, the MID-sub-group is characterized by the unique RWP-RK domain and includes algae and *Oryza sativa* members but not *Arabidopsis* or *Lotus japonicus* members, suggesting the loss of the RWP-RK domain in eudicot lineages. MID proteins are involved in gametogenesis in green algae and the regulation of sexual differentiation in bryophytes (Koi et al. 2016).

Genome-wide identification and phylogenetic analyses at a later time point on RKDs from both nodulating and non-nodulating plants have revealed surprising findings. The RWP-RK motif, previously believed to be plant-exclusive, is an ancient motif that predates the division between fungi and Plantae, challenging prior assumptions and providing new insights into the evolutionary history of RKDs (Schauser et al. 2005; Chardin et al. 2014). To understand the evolutionary basis of RKDs and NLPs further, a large-scale study involving 587 NLP proteins with RWP-RK and PB1 motifs from 74 plant species, as well as seven algae species without NLP proteins but some RWP-RK motifs, was conducted by Mu and Lu (2019). Their findings suggest that NLP proteins originated from green algae and were dispersed during the evolutionary history of plants. The study classified *A. thaliana* NLPs into three different groups, with *At*NLP1-5 evolving after the division of eudicots and monocots, while *At*NLP6–7 evolved from green algae, and *At*NLP8–9 appeared to evolve from mosses.

In a more recent study, Wu and colleagues (2020) analyzed twenty-six species belonging to the nitrogen-fixing clade (NFC), with and without nodulation capacity, investigating NLPs and RKDs. The researchers found that the RWP-RK domain is widely distributed among NFC species, whereas NLPs appear to be restricted to plants with nodulation capacity. Additionally, the RWP-RK domain was divided into eleven ortho-groups, suggesting diverse origins and supporting the idea that the divergence of the RWP-RK domain contributed to species adaptation to fluctuations in nitrogen environmental viability (Wu et al. 2020). In another study, Sakuraba and colleagues (2022) updated the phylogeny with eight different plant species belonging to the Viridiplantae kingdom, describing five monophyletic clades based on their unique characteristics. These studies collectively enhance our understanding of the evolution and functional significance of RWP-RK proteins and NLPs in the context of nitrogen sensing and signaling across various plant lineages.

Regarding functional roles, RWP-RK transcription factors play diverse roles in plant development and physiology, encompassing stress responses, light sensing, cell cycle regulation, and control of flowering time, among other functions (O´Brien et al. 2016; Yan et al. 2016; Liu et al. 2017; Zhang et al. 2021). These TFs are also key players in plant adaptation to environmental changes and stresses, as they respond to various stimuli, including changes in light intensity. NLP proteins have been identified as regulators of nitrogen use efficiency (NUE)-related genes, binding to promoters to modulate their expression (Chardin et al. 2014). In contrast, RKD genes are primarily involved in gametogenesis and embryogenesis processes (Konishi and Yanagisawa 2013a, b).

**Fig. 2:**
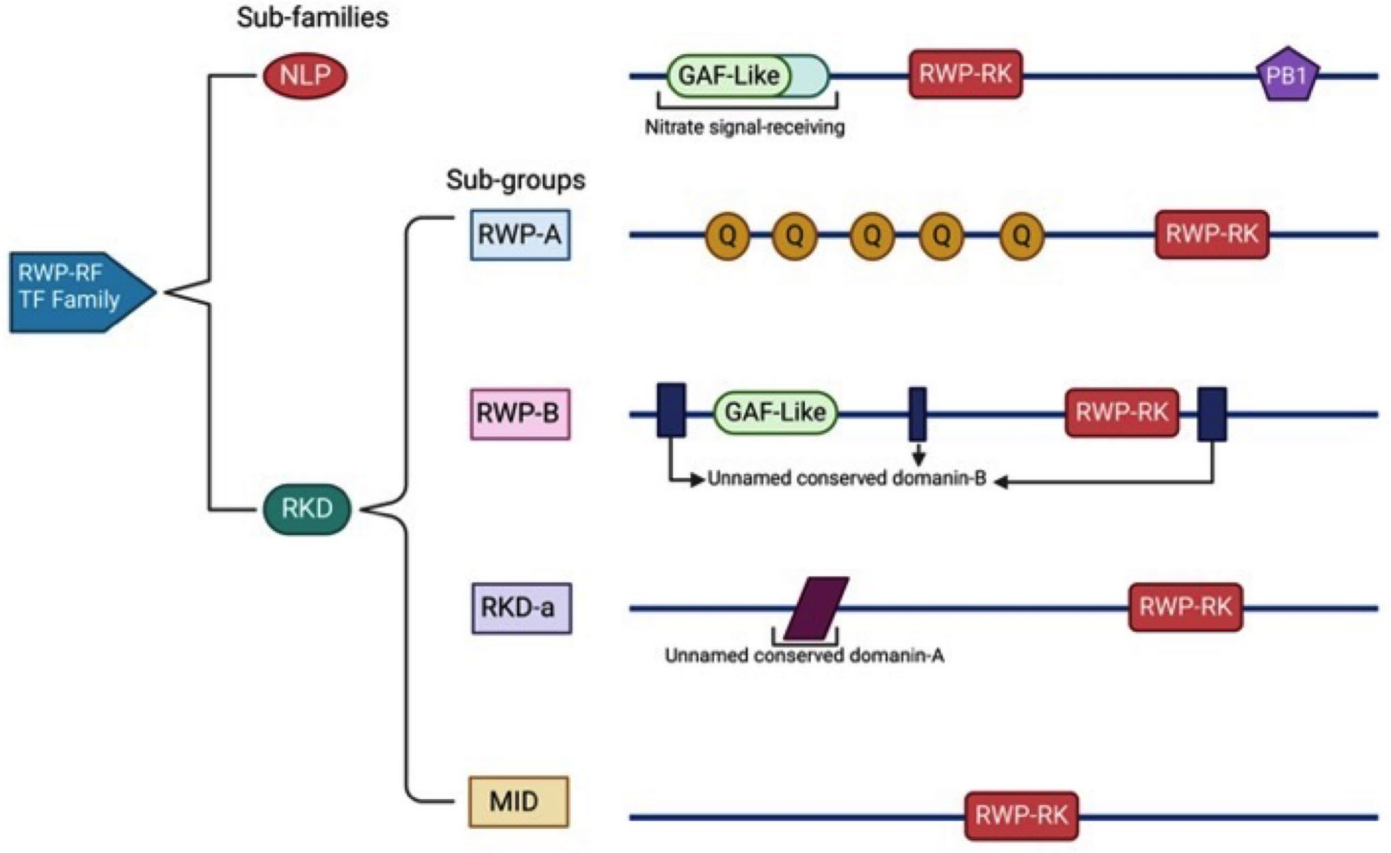
Classification of the RWP-RK family domains. An NLP sub-family is represented by one sub-group with three defined domains. On the other hand, the RKD sub-group is divided into four groups RWP-A, RWP-B, RKD and MID with different characteristics on each and unnamed conserved domains

The classification of RWP-RK proteins is often divided into two main groups, NLPs and RKDs. However, some authors adopt a more comprehensive classification approach. In this study, we analyze the ancestry, distribution, and classification of the RWP-RK domain across the Eukaryotic kingdom. Figure 2 presents the main distribution of the RWP-RK domain, identifying two sub-families and four sub-groups with their respective characteristic features. Table 1 provides information on 103 species within the Eukaryotic kingdom, detailing the number of proteins containing the RWP-RK domain present in each genome, categorized as NLPs and RKDs. Remarkably, the RWP-RK domain is found across a broad range of organisms, including algae, mosses, eudicots, monocots, and oomycetes, which were recently added to the list of proteins with this domain. Notably, Yin and coworkers (2020) have uncovered RWP-RK transcription factor homologs in oomycete pathogens, indicating shared sub-groups with plant-specific RWP-RKs. These findings suggest a potential evolutionary divergence from their plant counterparts. Moreover, the oomycete RWP-RKs have been associated with pathogenesis and virulence, indicating their involvement in host–pathogen interactions. In a recent study by Amin et al. (2023), RWP-RK transcription factors in soybean were identified and thoroughly characterized. They discovered a total of 70 RWP-RK transcription factors in the soybean genome and categorized them into nine sub-groups based on their phylogenetic relationships. Additionally, they identified conserved domains like RWP-RK, B2, and GRAS within the RWP-RK proteins. Table 2 shows the information on the domains and representative characteristics of the RWP-RK family classification.

**Table 1:** RWP-RK domains are distributed in the Eukarya kingdom

Lineage		Organism	Gene total number	NLPs number	RKDs number	References^#^
Eucaryota/ amoebozoa	Dictyosteliales	*Dictyostelium discoideum*	2	0	2	A, D
Algaes		*Chlamydomonas reinhardtii*	16	0	16	A, B, D, E
		*Chromochloris zofingiensis*	7	1	6	B
		*Coccomyxa subellipsoidea* C-169	0	0	6	B
Bronw algae	Phaeophyceae	*Ectocarpus siliculosos*	7	0	0	D*
		*Nannochloropsis gaditana*	1	0	0	D*
Chlorophyte		*Dunaliella salina*	6	1	5	B
		*Micromonas pusilla* CCMP1545	6	3	3	B
		*Micromonas sp* RCC299	6	2	4	B
		*Ostreococcus lucimarinus*	5	1	4	B
		*Porphyra umbilicalis*	3	1	2	B
		*Volvox carteri*	10	0	10	A, B, D
		*Zostera marina*	7	2	5	B
Mosses		*Marchantia polymorpha*	4	1	3	B, D, E
		*Physcomitrella patens*	13	7	6	B, D, E
		*Sphagnum fallax*	9	5	4	B
		*Anthoceros angustus*	10	6	4	E
		*Chara braunii*	5	0	5	E
Ferns		*Selaginella moellendorffii*	5	3	2	B
Oomycota	*Peronosporaceae*	*Phytophthora sojae*	18	0	0	D*
		*Phytium ultimum*	15	0	0	D*
Angiosperms	Amborellaceae	*Amborella trichopoda*	4	3	1	B
Eudicots	Amaranthaceae	*Amaranthus hypochondriacus*	6	6	0	B
	Anacardiaceae	*Anacardium occidentale*	7	6	1	B
	Apiaceae	*Daucus carota*	21	21	0	B
	Asteraceae	*Helianthus annuus*	32	31	1	B
		*Lactuca sativa*	10	9	1	B
	Brassicaceae	*Arabidopsis halleri*	10	9	1	B
		*Arabidopsis lyrata*	10	9	1	B
		*Arabidopsis thaliana*	14	9	5	A, B, C, D, E
		*Boechera stricta*	10	9	1	B
		*Brassica oleracea capitata*	6	5	1	B
		*Brassica rapa FPsc*	15	14	1	B
		*Capsella grandiflora*	10	9	1	B
		*Capsella rubella*	10	9	1	B
		*Eutrema salsugineum*	10	9	1	B
	Chenopodiaceae	*Chenopodium quinoa*	15	9	6	B
	Crassulaceae	*Kalanchoe fedtschenkoi*	12	10	2	B
		*Kalanchoe laxiflora*	24	20	4	B
	Cucurbitaceae	*Cucumis sativus*	4	3	1	B
		*Begonia fuchsioides*	22	15	7	C
		*Datisca glomerata*	9	5	4	C
	Fagales, Betulaceae	*Alnus glutinosa*	10	4	6	C
	Fagales, Casuarinaceae	*Casuarina glauca*	9	4	5	C
	Caricaceae	*Carica papaya*	2	2	0	B
	Euphorbiaceae	*Ricinus communis*	6	6	0	B
		*Manihot esculenta*	8	7	1	B
	Linaceae	*Linum usitatissimum*	14	14	0	B
	Fabaceae	*Arachis ipaensis*	17	8	9	C
		*Arachis duranensis*	15	8	7	C
		*Cajanus cajan*	15	7	8	C
		*Cercis canadiensis*	7	4	3	C
		*Chamaecrista fasiculata*	11	7	4	C
		*Cicer arietinum*	9	5	4	B, C
		*Glycine max*	28	13	15	B, C, D
		*Lotus japonicus*	10	6	4	B, C, E
		*Lupinus angustifolius*	3	1	2	C
		*Medicago truncatula*	12	6	6	A, B, C
		*Mimosa pudica*	10	4	6	B, C
		*Nissolia schottll*	10	5	5	C
		*Phaseolus vulgaris*	12	7	5	B, C
		*Trifolium pratense*	11	5	6	B, C
		*Trifolium subterraneum*	13	8	5	C
		*Vigna unguiculata*	8	7	1	B
		*Vigna angularis*	7	1	6	C
		*Vigna radiata*	10	6	4	C
	Malvaceae	*Theobroma cacao*	5	4	1	B
		*Gossypium raimondii*	12	11	1	B
		*Gossypium hirsutum*	22	20	2	B
		*Eucalyptus grandis*	5	5	0	B
	Oleaceae	*Olea europaea*	12	11	1	B
	Rosaceae	*Fragaria vesca*	4	3	1	B
		*Malus domestica*	7	5	2	D
		*Prunus persica*	5	4	1	B
		*Dryas drummondii*	8	4	4	C
		*Discaria trinervis*	8	4	4	C
		*Trema Tomentosa*	8	4	4	C
		*Parasponia adersonii*	8	4	4	C
	Ranunculaceae	*Aquilegia coerulea*	5	3	2	B
	Rutaceae	*Citrus sinensis*	4	3	1	B
		*Citrus clementina*	5	4	1	B
	Solanaceae	*Nicotiana tabacum*	1	0	1	D
		*Solanum lycopersicum*	6	6	0	B
		*Solanum tuberosum*	1	12	0	B
	Scrophulariaceae	*Mimulus guttatus*	9	9	0	B
	Salicaceae	*Populus deltoides WV94*	13	10	3	B
		*Populus trichocarpa*	16	14	2	B
		*Salix purpurea*	14	13	1	B
	Vitaceae	*Vitis vinifera*	5	4	1	B
Monocots						
	Bromeliaceae	*Ananas comosus*	10	8	2	B
	Liliaceae	*Asparagus officinalis*	3	3	0	B
	Lemnaceae	*Spirodela polyrhiza*	5	4	1	B
	Musaceae	*Musa acuminata*	11	11	0	B
	Poaceae	*Brachypodium distachyon*	16	7	9	A, D
		*Brachypodium hybridum*	15	13	2	B
		*Brachypodium stacei*	7	7	0	B
		*Brachypodium sylvaticum*	8	7	1	B
		*Hordeum vulgare*	6	5	1	B
		*Miscanthus sinensis*	12	10	2	B
		*Oropetium thomaeum*	6	5	1	B
		*Oryza sativa*	15	5	10	A, E
		*Panicum hallii*	7	6	1	B
		*Panicum virgatum*	14	12	2	B
		*Setaria italica*	9	7	2	B
		*Setaria viridis*	8	7	1	B
		*Sorghum bicolor*	5	5	0	B
		*Triticum aestivum*	24	18	6	B, D
	Poales	*Zea mays*	10	9	1	B

^#^References: Chardin et al. (2014); Mu and Lu (2019); Wu et al. (2020); Yin et al. (2020); Sakuraba et al. (2022)

**Table 2:**
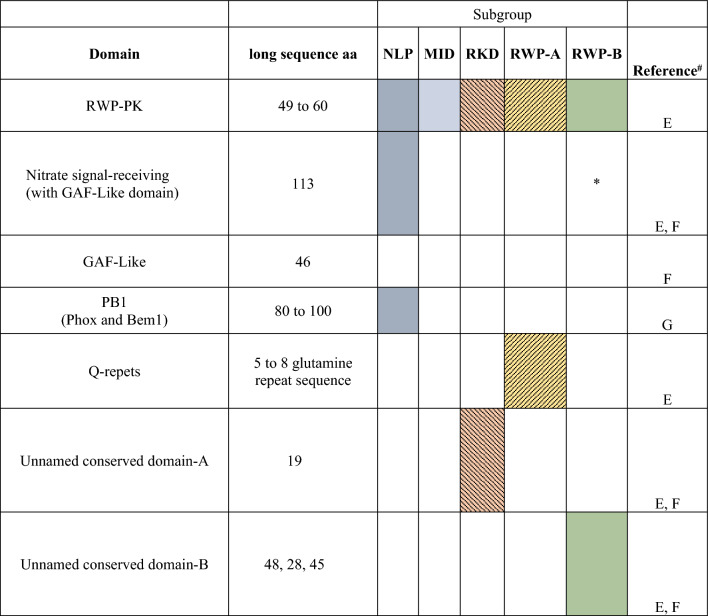
Characteristics of the RWP-RK family according to NLPs and RKD sub-groups

*The GAF-Like domain is present only in some of the members of the RWP-B sub-group

^#^References: Sakuraba et al. (2022); Bailey et al. (2015); Mutte and Weijers (2020)

Overall, the classification and analysis of RWP-RK proteins shed light on their diverse functional roles and evolutionary history within the eukaryotic kingdom. Further research in this area will undoubtedly deepen our understanding of these essential TFs and their significance in various biological processes.

## NLPs are nitrate responsive transcription factors

Nitrates play a significant role in plant growth and development, serving as vital signaling molecules involved in the primary nitrate response (PNR) (Gowri et al. 1992; Liu et al. 2017). Nitrate represents the most abundant nitrogen source in aerobic soils (Fan et al. 2017) and is the major form of nitrogen absorbed by crops. Alongside ammonium and certain organic molecules like amino acids, nitrate acts as both a nutrient and a signaling molecule, influencing various cellular processes, including gene expression regulation, plant growth, morphogenesis, lateral root development, flowering, seed dormancy release, and auxin transport (Matakiadis et al. 2009; O’Brien et al. 2016; Zhang et al. 2019, 2021; Fredes et al. 2019; Vidal et al. 2020).

**Fig. 3:**
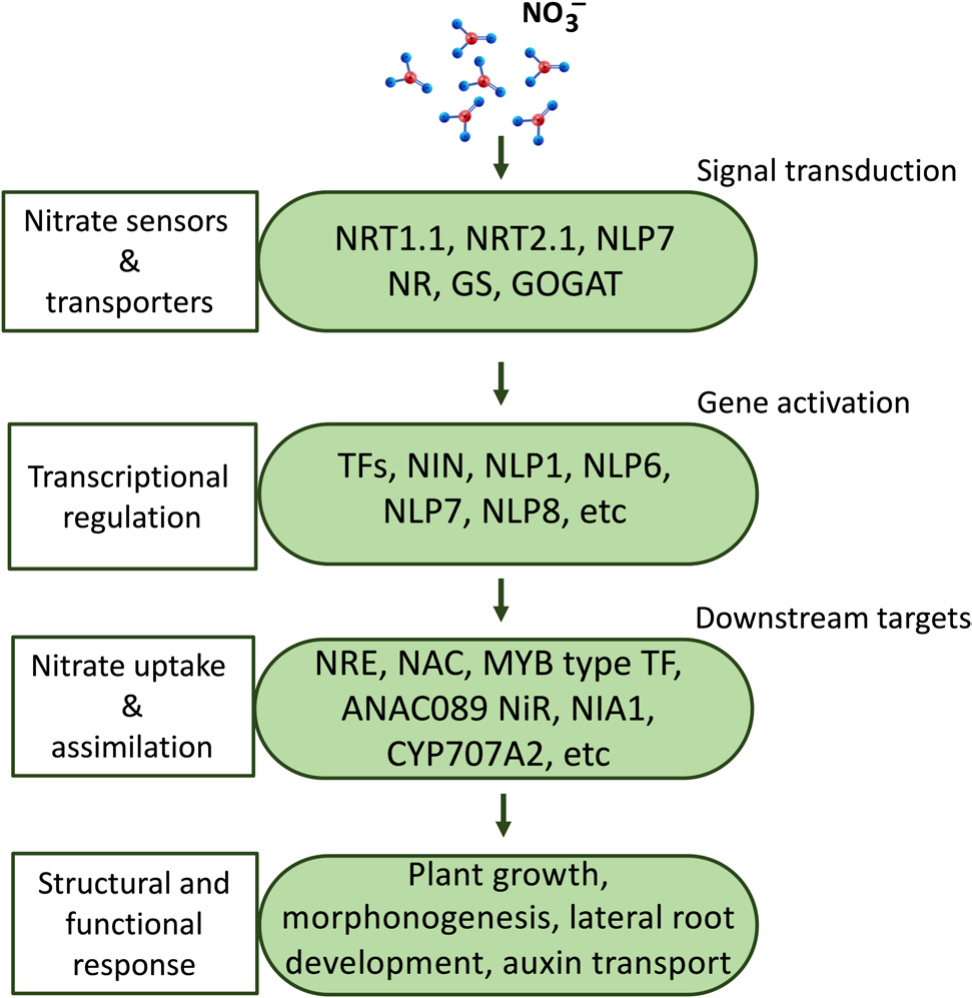
Illustrating an overview of plant responses to nitrate, encompassing the activation of nitrate sensors, subsequent transport to the cytoplasm, activation, and transcriptional regulation of genes. This cascade leads to downstream processes including nitrate uptake and assimilation, culminating in structural and functional responses within plants

The primary nitrate response involves the participation of various genes, such as nitrate sensors like NRT1.1, *NITRATE REDUCTASES* (*NR*), *NITRITE REDUCTASES* (*NiR*), *GLUTAMINE SYNTHASE* (*GS*), and *GLUTAMATE SYNTHASE* (*GOGAT*) (Krapp et al. 2014). Among these, the NIN-like protein transcription factors (TFs) have emerged as crucial players in PNR (Fig. 3). Molecular evidence from *Arabidopsis* transgenic lines expressing NLP6 promoter reveals specific β-*GLUCURONIDASE* (GUS) induction in response to nitrate (Konishi and Yanagisawa 2013a, b), strongly suggesting NLPs role as nitrate-responsive TFs. Additionally, extensive research has explored the nitrate responsiveness of NLPs in various plants. In *Arabidopsis*, nine NLPs (*At*NLP1–*At*NLP9) have been identified, all capable of binding to the *NITRATE-RESPONSIVE CIS-ELEMENT* (NRE) and activating NRE-dependent nitrate-responsive gene expression (Konishi and Yanagisawa 2013a, b). Similarly, in the legume *L. japonicus*, all five NLPs, including NIN, have been shown to directly bind to the NRE motif, promoting the transcription of target genes (Suzuki et al. 2013). However, while the amino-terminal regions of *Lj*NLP1 and *At*NLP’s respond to nitrate signaling, the N-terminal region of NIN does not exhibit a nitrate response. Interestingly, NIN has been found to regulate nitrate-induced gene expression antagonistically in *L. japonicus* (Soyano et al. 2015).

NLPs serve as major transcription factors governing diverse physiological responses in plants dependent on nitrate levels. However, they likely collaborate with other transcription factors to impart specificity to each response. In *A. thaliana* for example, NLP7 and NLP8 have been shown to regulate the expression of genes involved in N uptake and assimilation, and their overexpression leads to increased biomass and N use efficiency (Konishi and Yanagisawa 2010; Li et al. 2010). Specifically, NIN-like protein 7 (NLP7) loss-of-function mutants in *Arabidopsis* display a nitrate-starvation phenotype even under nitrate-sufficient conditions. Notably, *Arabidopsis* NLP7 is retained in the nucleus upon CPK-dependent phosphorylation at residue S205 in response to nitrate, where it directly binds to nitrate response genes, thus regulating the PNR (Marchive et al. 2013). This induction of NLP7 is rapid and specific to nitrate, showing no response to NH_4_^+^ (Medici et al. 2015). NLP7's function is directly modified by NO_3_^−^, playing a crucial role in PNR, and it is also required to achieve the maximal expression level of N-responsive genes in an N-rich environment. NLP7 is further implicated in the NO_3_^−^-dependent induction of NO_3_^−^ transport and assimilation genes like NRT2.1, NRT2.2, and NIA1. Moreover, NLP7 physically interacts with NRG2 in the NRT1.1 pathway and regulates the transcription levels of numerous genes involved in N sensing, transport, and assimilation (Castaings et al. 2009; Marchive et al. 2013; Xu et al. 2016).

NLP7 functions as a master switch, which controls the expression of a large number of genes in response to nitrate changes by binding to the promoter region of NRT1.1 to regulate its activity (Zhao et al. 2018; Liu et al. 2017). A recent finding from Liu et al. (2022) identified AtNLP7as the primary nitrate sensor and indicates that NLP7 is derepressed upon nitrate perception via its N terminus. Transcriptome reprogramming in primary nitrate responses triggered by nitrate was abolished in NLP7 mutant. NLP7 is found to have dual regulatory modes as a transcription activator and as an intracellular nitrate sensor. While NRT1.1 functions as a plasma membrane bound extracellular nitrate transceptor, NLP7 was found to be a distinct nitrate sensor (Castaings et al. 2009; Ho et al. 2009; Wang et al. 2009; Konishi & Yanagisawa 2010). In addition to its pivotal role as a master regulator, NLP7 is also a commander, directly controlling downstream elements of N transduction pathways.

Another member of the NLP family, NLP8, plays a critical role in orchestrating transcriptional reprogramming for NO_3_^−^-dependent seed germination. However, unlike NLP7, NLP8's regulation does not rely on nitrate-dependent nuclear retention, as it remains in the nucleus regardless of nitrate application (Yan et al. 2016). NLP8 is constitutively localized in the nucleus and significantly contributes to nitrate-promoted seed germination in *Arabidopsis*. It regulates well-known NO_3_^−^-sensitive genes like NIA1 or NIR, as well as specific targets such as the ABA catabolic gene CYP707A2. Furthermore, NLP2, akin to other NLP family members, is rapidly induced by nitrate and has been identified as a nitrate sensor in *Arabidopsis* (Durand et al. 2023). NLP2 plays a crucial role in nitrate-dependent regulation of carbon and energy-related processes, potentially influencing plant growth under different nitrogen environments. Additionally, NLP2 regulates vegetative growth, as evidenced by reduced rosette biomass in both *nlp2* and *nlp7* single mutants grown in the presence of nitrate (Konishi et al. 2021). Although NLP2 and NLP7 share similar properties such as nitrate-dependent activation, null mutants of NLP2 cannot be rescued by supplementation with ammonium or glutamine (Konishi et al. 2021). This suggests that the growth defects in *nlp2* mutants may arise from additional nitrate-controlled processes beyond nitrate uptake and assimilation. NLP2 is instrumental in orchestrating nitrogen assimilation and metabolic pathways, ensuring a steady supply of carbon skeleton and energy in response to soil nitrate availability (Durand et al. 2023).

NLP2, alongside its role in nitrate sensing, has been identified to play a crucial part in the regulation of root development and lateral root growth in response to changes in nitrate availability (Konishi et al. 2021). Additionally, the regulation of NLP2 activity by nitrate has been found to involve a signaling pathway that includes the production of nitrate reductase-associated nitric oxide (NO) in rice, which plays a role in regulating salt stress during seed germination by ABA catabolism (Yi et al. 2022).

The transcriptome analysis of the PNR has revealed that NLP6 and NLP7 collectively control approximately 50% of nitrate-induced genes, exhibiting two distinct expression patterns based on cluster analysis. Moreover, beyond their involvement in nitrate signaling, NLP6 and NLP7 have also been shown to participate in high ammonium conditions. Growth phenotypes and transcriptome data suggest that NLP6 and NLP7 are fully functionally redundant and may act as repressors in response to ammonium (Cheng et al. 2023). *At*NLP6 is found to be involved in nitrate activate transcription specifically, in the regulation of nitrate reductase genes such as NIA1 and NIR1 genes, through a conserved motif named NRE-43-d8 (Suzuki et al. 2013). A similar mechanism of action was reported in maize by Liseron-Monfils et al. (2013). Remarkably, when *Zm*NLP6 and *Zm*NLP8 were overexpressed in the *Arabidopsis* nitrate regulatory gene mutant NLP7, they successfully recovered nitrate assimilation and induction of nitrate-responsive genes to wild-type (WT) levels, indicating their ability to replace the essential roles of the master nitrate regulatory gene *At*NLP7. These genes, *Zm*NLP6 and *Zm*NLP8, were found to be localized in the nucleus and capable of binding candidate nitrate-responsive *cis*-elements in vitro (Cao et al. 2017). Additionally, the phylogenetic relationship analysis of NLPs in maize revealed that *Zm*NLP5 shows a closer relation to *At*NLP7 and is significantly associated with 39 genes involved in nitrogen assimilation, response, and signaling in maize plants, suggesting a pivotal role of *Zm*NLP5 under nitrogen limitation (Ge et al. 2020). Furthermore, studies on *Zm*NLP3 demonstrated its involvement in nitrate signaling response and assimilation processes (Wang et al. 2018).

In *M. truncatula*, NLP1 plays a critical role in mediating nitrate-induced inhibition of nodulation. Specifically, NLP1 directly binds to the CLE35 promoter, activating its expression and enabling nitrate-mediated inhibition of nodulation in autoregulation of nodulation and NLP1-dependent manner (Lebedeva et al. 2021; Mens et al. 2021; Moreau et al. 2021). Similarly, NLP1 and NLP5 are rapidly and temporarily induced by nitrate in tomatoes (Liu et al. 2021).

## NLPs in symbiosis

NIN-like proteins constitute a crucial family of transcription factors that exert significant influences on various aspects of plant development and their responses to environmental stimuli, including interactions with beneficial microorganisms like rhizobia and mycorrhizal fungi (Markmann and Parniske 2009; Doyle 2011; Pawlowski and Demchenko 2012; Liu and Bisseling 2020). These symbiotic microorganisms form mutualistic associations with plants, facilitating the exchange of nutrients and resources, ultimately benefiting both partners. In leguminous plants, NIN and NIN-like genes play indispensable roles in the initiation and development of nitrogen-fixing nodules. These genes are particularly involved in the early stages of nodule formation, encompassing crucial processes like the establishment of infection threads, cortical cell divisions, and activation of symbiotic gene expression in both the plant and the rhizobial partner (Marsh et al. 2007; Liu and Bisseling 2020). Notably, loss-of-function studies in several legumes have shown that the absence of NIN leads to excessive root hair curling and complete cessation of infection thread formation (Schauser et al. 1999; Batagov et al. 2003; Marsh et al. 2007). Additionally, during *Frankia*-induced root hair deformation in *Casuarina glauca*, NIN has been demonstrated to be essential for this process (Clavijo et al. 2015).

While NIN and NIN-like genes exhibit conservation across various legume species, differences in their sequences and expression patterns suggest possible functional divergence in different legume lineages. It is plausible that certain legumes possess multiple copies of NIN-like genes, each serving specialized functions in distinct stages of nodule development or response to various environmental cues. The evolutionary trajectory of NIN and NIN-like genes may have been shaped by the necessity to establish and maintain effective symbiotic relationships with specific rhizobial strains, as well as environmental factors influencing nodule development (Liu and Bisseling 2020). Research has shed light on the regulatory role of NLPs in governing the expression of genes critical for establishing and sustaining these symbiotic associations. For instance, in legumes like *L. japonicus* and *M. truncatula*, NLPs have been found essential for the formation of root nodules accommodating nitrogen-fixing bacteria (Liu and Bisseling et al. 2020). Furthermore, NLPs have been implicated in the establishment of mycorrhizal associations, facilitating nutrient access, particularly phosphorus, for plants (Clavijo et al. 2015; Bu et al. 2019). Genetic investigations focusing on *L. japonicus* and *Medicago* have elucidated the pivotal roles of NLPs in governing root nodule formation in response to nitrate. In the context of *L. japonicus*, *NITRATE UNRESPONSIVE SYMBIOSIS 1* (NRSYM1), while not directly induced by nitrate, exhibits nuclear localization influenced by nitrate levels. NRSYM1 was identified as the gene encoding *Lj*NLP4 through forward genetic screening that specifically targeted nitrate-induced control of root nodule symbiosis. *Lj*NLP4 plays a critical role in various aspects of root nodule symbiosis triggered by nitrate, including nodule organogenesis and mature nodule activity. Nitrate acts as a trigger for the nuclear accumulation of *Lj*NLP4, enabling its binding to the promoter of CLE-RS2 in a nitrate-dependent manner, consequently inducing CLERS2 expression (Nishida et al. 2018). Additionally, research exploring NRSYM1/*Lj*NLP4 and NRSYM2/*Lj*NLP1 revealed their overlapping functions as master regulators, orchestrating nitrate-dependent gene expression in *L. japonicus* (Nishida et al. 2021).

In the soybean genome, four homologs of *At*NLP7, designated as *Gm*NLP7a–*Gm*NLP7d, have been identified, all of which display responsiveness to nitrate while remaining unresponsive to rhizobia. Downregulation of *Gm*NLP7s resulted in an increased number of nodules, whereas overexpressing *Gm*NLP7a (*Gm*NLP7aOE) led to a reduction in nodule number regardless of nitrate presence. These findings imply that *Gm*NLP7s function as negative regulators in the nodulation process. Interestingly, the overexpression of *Gm*NLP7a did not result in increased nitrogenase activity, but it did downregulate the expression of *Gm*NIN1a and GmENOD40. Further investigations uncovered the interaction between *Gm*NLP7a and *Gm*NIN1a through the PB1 domain (Wu et al. 2023). In the context of *Medicago*, the presence of nitrate initiates the translocation of *Mt*NLP1 from the cytosol to the nucleus, where it engages with *Mt*NIN through the PB1 domain, resulting in the repression of *Mt*NIN's activation on *Mt*CRE1, a crucial player in nodule organogenesis. Additionally, *Mt*NLP1 exerts its regulatory influence by directly binding to the promoter region of *Mt*CLE35, thus triggering the expression of this gene (Lin et al. 2018). These findings underscore the pivotal role of *Mt*NLP1 as a key molecular component responsible for mediating the nitrate-dependent control of root nodule symbiosis in *Medicago*. Furthermore, in response to nitrate, the expression of *Mt*NRT2.1 is upregulated, and this process occurs in a *Mt*NLP1-dependent manner (Lin et al. 2018). However, recent research by Luo et al. (2023) revealed that under low-nitrate conditions, the translocation of *Mt*NLP1 to the nucleus is significantly reduced. Intriguingly, the impact of nitrate uptake by *Mt*NRT2.1 on nodulation varies depending on the nitrate levels present. Under low-nitrate conditions, the interplay between *Mt*CEP1 and *Mt*NLP1 plays a pivotal role in regulating nodulation responses. These findings provide valuable insights into the intricate mechanisms of nitrate signaling and its influence on the nodulation process in plants (Luo et al. 2021).

## NLPs in abiotic stress

NLPs have been shown to play important roles in plant responses to various abiotic stresses, such as drought, salinity, and cold (Castaings et al. 2009; Yi et al. 2022; Ding et al. 2022). In *Arabidopsis*, for example, the expression of several NLP genes is induced by drought, and overexpression of some NLP´s can enhance drought tolerance by increasing water use efficiency and reducing water loss. NLPs have also been implicated in cold stress responses in plants (Ding et al. 2022). In *Arabidopsis*, some members of NLP genes are induced by cold, and overexpression of them has been shown to enhance cold tolerance. Calcium-dependent protein kinase CPK28 gets activated leading to the phosphorylation of NLP7 in the cytoplasm. This phosphorylation facilitates NLP7's rapid nuclear translocation and orchestrates transcriptional reprogramming of cold-responsive genes, enabling the plant to adapt to cold conditions. Interestingly, NLP7 shares common target genes in nitrate and cold stress responses, suggesting a potential link between nitrogen and cold signaling (Ding et al. 2022).

NIN-Like Proteins play pivotal roles in plant responses to abiotic stresses and are also involved in intricate crosstalk between different stress signaling pathways. Studies in *Arabidopsis* have revealed that NLPs interact with proteins linked to both abiotic and biotic stress responses, suggesting their involvement in coordinating plant defenses against multiple stresses (Karve et al. 2016; Guan 2017; Hartman 2021; Liu et al. 2022; Ding et al. 2022). In the context of indica rice, a subspecies of *Oryza sativa*, research focusing on gene expression profiles identified 17 NIN-Like genes associated with the early response phase to chilling stress. These transcription factors likely participate in stress signaling, antioxidant defense, osmotic regulation, and other mechanisms to enable rice seedlings to cope with chilling stress (Pradhan et al. 2019). A genome-wide analysis in rice led to the discovery of 10 NLP genes, some of which were significantly induced under nitrogen stress conditions (Jagadhesan et al. 2020). Functional analysis of *Os*NLP3 and *Os*NLP10 through overexpression and knockdown experiments demonstrated their roles in enhancing and reducing nitrogen stress tolerance, respectively. Further transcriptomic analysis revealed downstream target genes associated with nitrogen uptake, assimilation, and signaling pathways, contributing to a comprehensive understanding of NLP-mediated responses to nitrogen stress.

Intriguingly, *Os*NLP2 was found to negatively regulate ferroptotic cell death, which may have implications for rice's response to the pathogen *Magnaporthe oryzae* (Chen et al. 2022). The study revealed that *Os*NLP2 interacted with the pathogen and influenced defense mechanisms, including the activation of immune-related genes, signaling pathways, and cellular responses during *M. oryzae* infection. On the other hand, another study describes the induction by salt stress of *Os*NLP2 that activates de abscisic acid (ABA) catabolism during seed germination (Yi et al. 2022). *Os*NLP1 was shown to improve yield and NUE in rice by regulating gene expression related to nitrogen metabolism, nutrient uptake, hormonal signaling pathways, and other physiological processes (Alfatih et al. 2020). Understanding the role of *Os*NLP1 in the response to nitrogen deficiency and its impact on plant growth and productivity is crucial for developing improved rice varieties with enhanced nutrient use efficiency. Another essential finding was the pivotal role of *Os*NLP4 in regulating NUE in rice, making it a promising candidate gene for enhancing crop yield and NUE (Wu et al. 2021). Additionally, a study demonstrated the functional interaction between NLP7 and PROTEOLYSIS6 (PRT6), which enhances tolerance to sucrose, ABA, and submergence stress (Castillo et al. 2021). NLP7 and PRT6 were found to modulate each other's activity and significantly influence the plant's response to sucrose, ABA, and submergence stress. Furthermore, NLPs are known to regulate stress-responsive genes by binding to specific DNA sequences in gene promoters. They can also interact with other transcription factors and co-regulators, influencing gene expression in response to stress signals. Post-translational modifications of NLPs further affect their cellular activity, stability, and localization. The study in soybean revealed that the expression analysis of RWP-RKs showed tissue-specific and stress-responsive patterns. Some RWP-RKs were specifically expressed in particular tissues, while others were induced by salt and heat stress. These insights provide a valuable understanding of the roles of RWP-RK transcription factors in soybean growth, development, and response to stress conditions (Amin et al. 2023).

## NLPs in other pathways and functions

​​ In addition to their involvement in abiotic stress response and nitrogen use efficiency, NLPs have been extensively investigated for their diverse roles in various biological pathways. For instance, nitrate, a crucial signaling component during root development, influences cell divisions at the root apical meristem and subsequent cell cycle progression (Guan et al. 2017; Gigli-Bisceglia et al. 2018). Plant growth-promoting rhizobacteria (PGPR) treatments also exhibit similar effects on root architecture through hormonal crosstalk (Verbon and Liberman 2016). Moreover, studies by Hernandez-Reyes et al. (2022) have shown that Rhizobium-induced signaling affects cell cycle progression and root cell elongation in an NLP5-dependent manner by inducing the expression of cell cycle-related cyclin D3 (CYCD3;1), a meiotic G1/S phase marker of the cell cycle. This indicates the role of NLP5 in cell proliferation and elongation patterns at the root tip.

The impact of NLPs on developmental processes, such as root development and flowering time, has also been explored. For example, *At*NLP6/7 has been demonstrated to regulate root meristem size and lateral root formation (Guan et al. 2017; Konishi et al. 2021), while *At*NLP7 and AtNLP3 have been implicated in flowering time and flower development regulation, respectively (Chardon et al. 2014; Xu et al. 2016). Additionally, NLP7 has been found to play a crucial role in controlling border-like cell release, a vital process for plant nutrient acquisition, by modulating gene expression involved in cell wall modification and degradation, including pectin degradation. NLP7 acts as a suppressor of genes like pectin lyase and CEL5 (CELLULASE5) (Karve et al. 2016). The absence of functional NLP7 in mutant plants results in reduced lateral roots and diminished nutrient uptake compared to wild-type plants, indicating the potential for improving nutrient acquisition in crop plants through genetic manipulation.

Furthermore, NLPs have been identified to have a novel function in *A. thaliana*, participating in chloroplast formation. They regulate the expression of genes involved in chloroplast biogenesis and their expression is influenced by developmental signals and environmental cues that control chloroplast biogenesis (Zhang et al. 2018). NLPs also act as sensors of the chloroplast's metabolic status, responding to the redox state of the organelle. Studies have also focused on the role of NLPs in plant–microbe interactions. For instance, in rice, the NLP transcription factor *Os*NLP6 is involved in the regulation of mycorrhizal symbiosis (Chen et al. 2022). In *M. truncatula*, the NLP *Mt*NIN plays a key role in the formation and function of root nodules in response to rhizobia infection (Schauser et al. 1999).
